# Supplementary material for: DNA methylation changes during acute COVID-19 are associated with long-term transcriptional dysregulation in patients’ airway epithelial cells
Source: EMBO Mol Med. 2025 Mar 21;17(5):923–37. doi: 10.1038/s44321-025-00215-5 (PMC12081608; doi:10.1038/s44321-025-00215-5)
Supplement: Supplementary file 12 — Appendix [file 44321_2025_215_MOESM12_ESM.pdf]

Appendix Figures for the Manuscript

DNA Methylation Changes During Acute COVID-19 are Associated with Long-Term Transcriptional Dysregulation in Patients' Airway Epithelial Cells

Marey Messingschlager, Sebastian D. Mackowiak, Maria Theresa Voelker, Matthias Bieg, Jennifer Loske, Robert Lorenz Chua, Johannes Liebig, Sören Lukassen, Loreen Thürmann, Anke Seegebarth, Sven Twardziok, Daria Doncevic, Carl Herrmann, Stephan Lorenz, Sven Klages, Fridolin Steinbeis, Martin Witzenrath, Florian Kurth, Christian Conrad, Leif E. Sander, Naveed Ishaque, Roland Eils, Irina Lehmann, Sven Laudi, Saskia Trump

Table of Contents

Appendix Figure S1……………………………………………………………………………..Page 2
Appendix Figure S2……………………………………………………………………………..Page 3
Appendix Figure S3……………………………………………………………………………..Page 4
Appendix Figure S4……………………………………………………………………………..Page 5
Appendix Figure S5……………………………………………………………………………..Page 6
Appendix Figure S6……………………………………………………………………………..Page 7
Appendix Figure S7……………………………………………………………………………..Page 8
Appendix Figure S8……………………………………………………………………………..Page 9
Appendix Figure S9 ……………………………………………………………………………Page 10
Appendix Figure S10 …………………………………………………………………………..Page 11

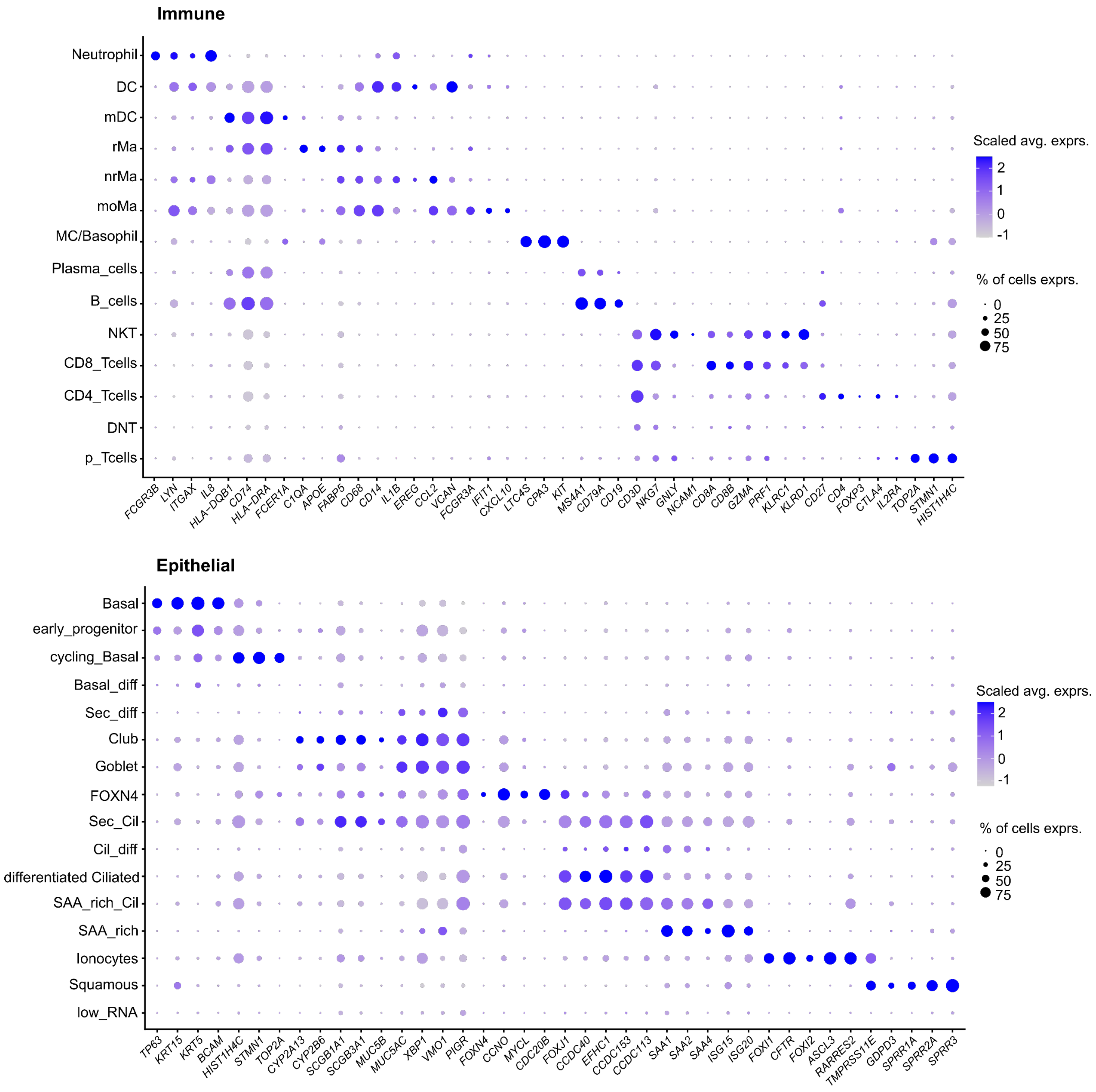

Appendix Figure S1: Dot plots showing the expression of cell type marker genes across immune and epithelial cells. DC = dendritic cells, mDC = myeloid dendritic cells, rMa = resident macrophages, nrMa = non-resident macrophages, moMa = monocyte-derived macrophages, MC/Basophil = mast cells or basophils, NKT = natural killer T cells, DNT = double negative T cells, p_Tcells = proliferating T cells, Basal_diff = differentiating basal cells, Sec_diff = differentiating secretory cells, FOXN4 = FOXN4^{+} cells, Sec_Cil = secretory-ciliated cells, Cil_diff = differentiating ciliated cells, SAA_rich_Cil = SAA^{+} ciliated cells, SAA_rich = SAA^{+} cells.

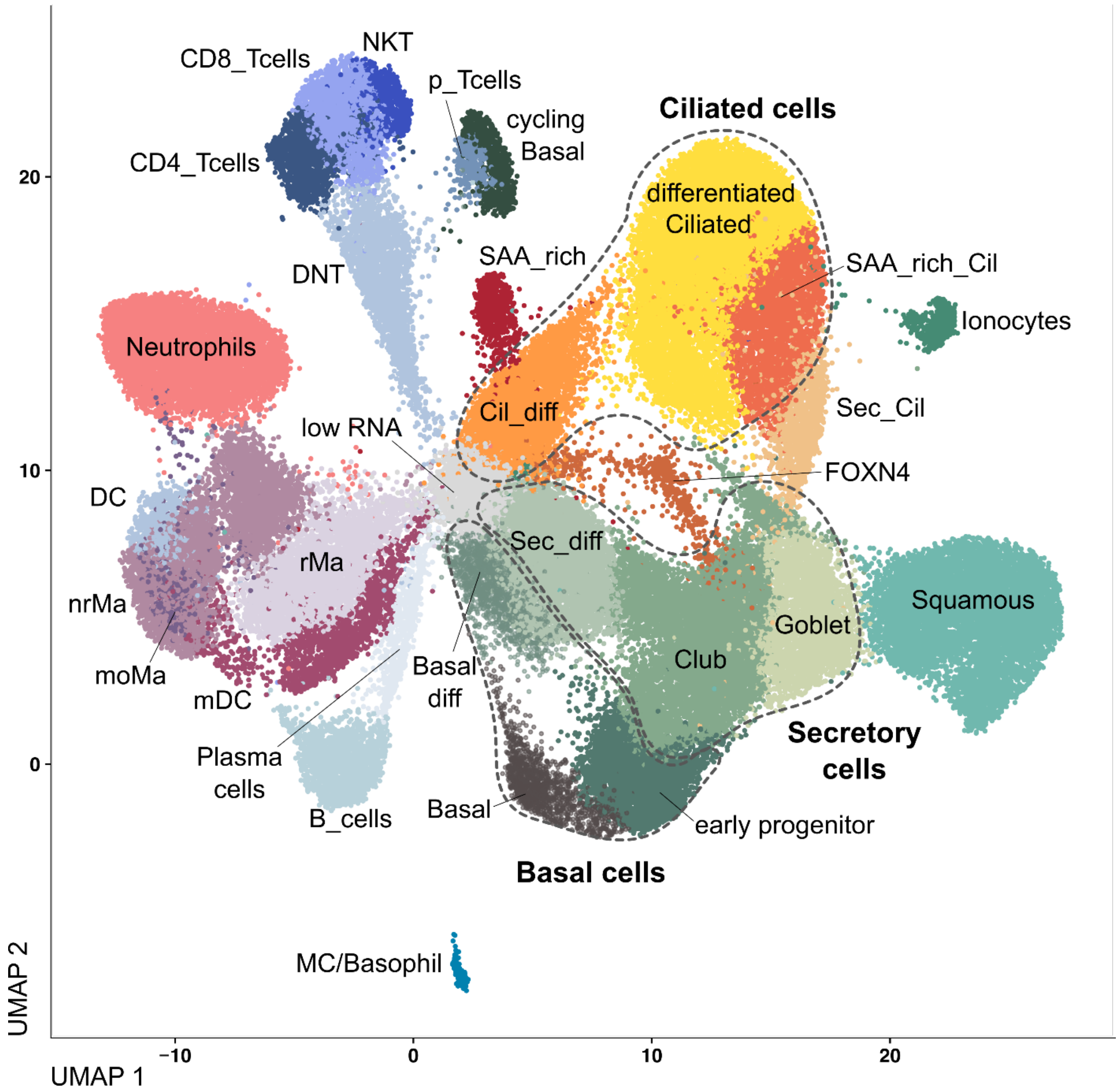

Appendix Figure S2: Uniform Manifold Approximation and Projection (UMAP) of 82,365 cells captured with scRNA-seq of nasal swabs from controls (n=10), SARS-CoV-2 infected patients (n=14) and post-infection follow-up samples from 3 and 12 months post-infection (n=12). For downstream analyses, differentiated Ciliated, Cil_diff and SAA_rich_Cil were grouped into **Ciliated cells**; Basal, Basal_diff and early progenitor into **Basal cells** and Sec_diff, Club and Goblet were summarized as **Secretory cells**.

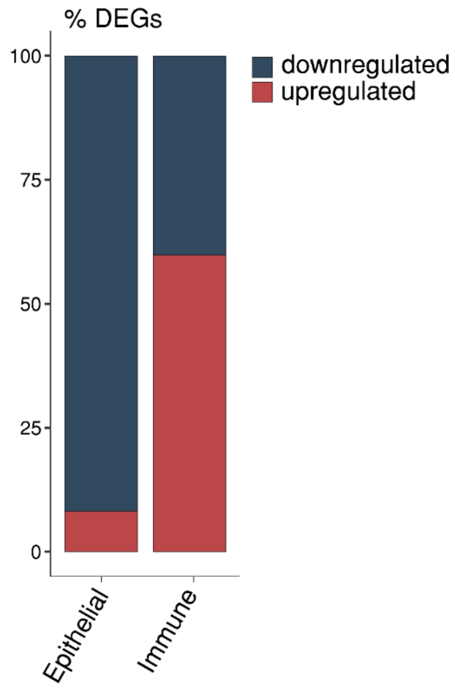

Appendix Figure S3: Percentage of up- and downregulated genes (from **Dataset EV2**) within all immune or epithelial DEGs. Differential gene expression was calculated using the MAST test, adjusted for age, sex and proportion of genes per cell.

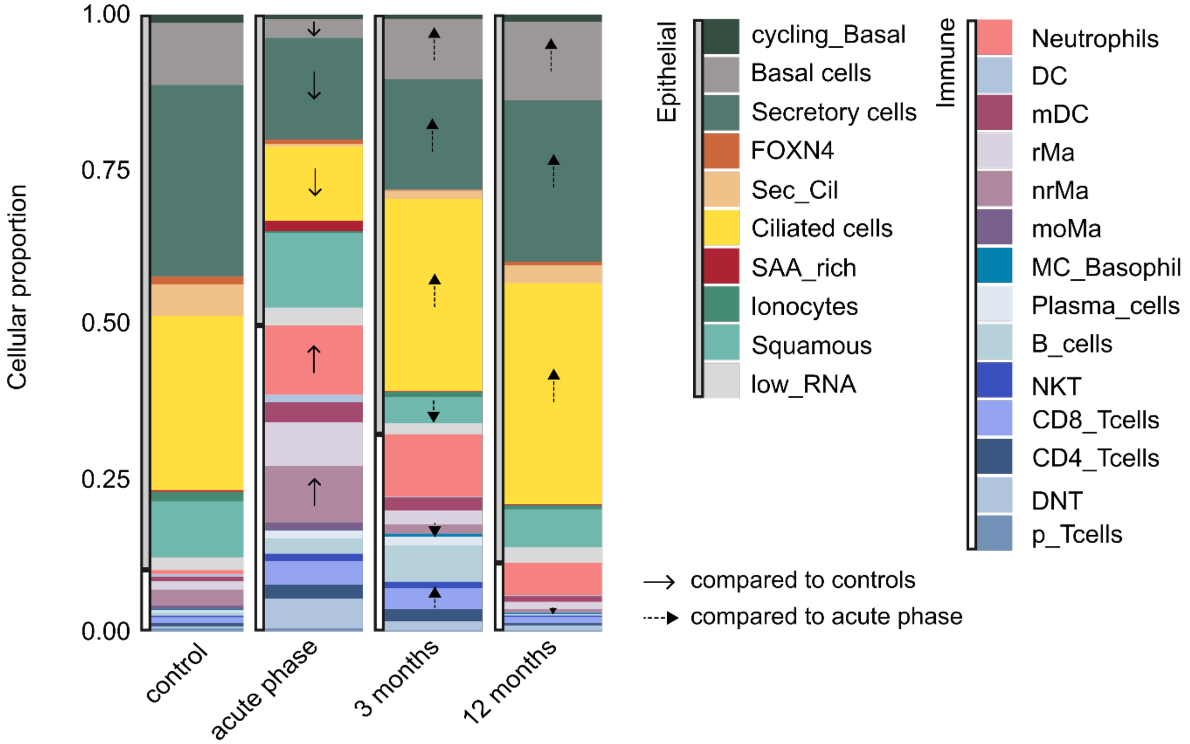

Appendix Figure S4: Cell type proportions in the scRNA-seq data across time points. Arrows indicate significant increases or decreases between groups (FDR-adj. $p<0.05$) as determined by scCODA (**Table EV6**). Ciliated cells = Ciliated, Cil_diff, SAA_rich_Cil, Basal cells = Basal, Basal_diff, early_progenitor, Secretory cells = Club, Goblet, Sec_diff

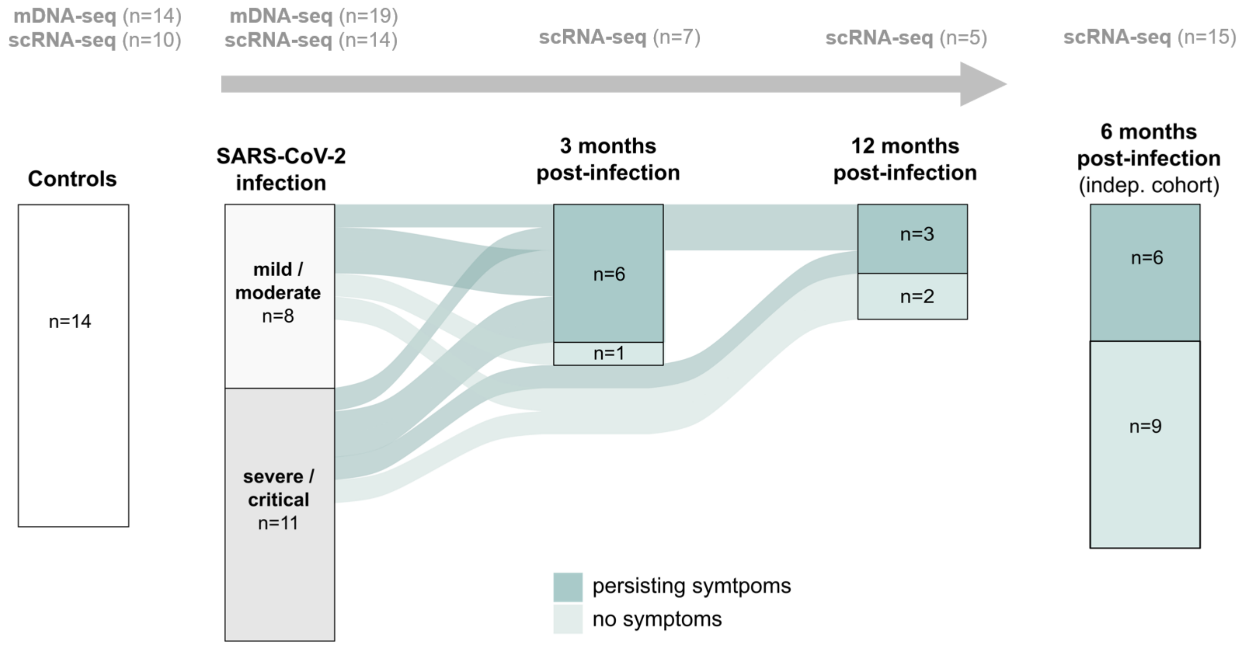

Appendix Figure S5: Sample overview and proportions of patients with or without persisting symptoms within the follow-up samples.

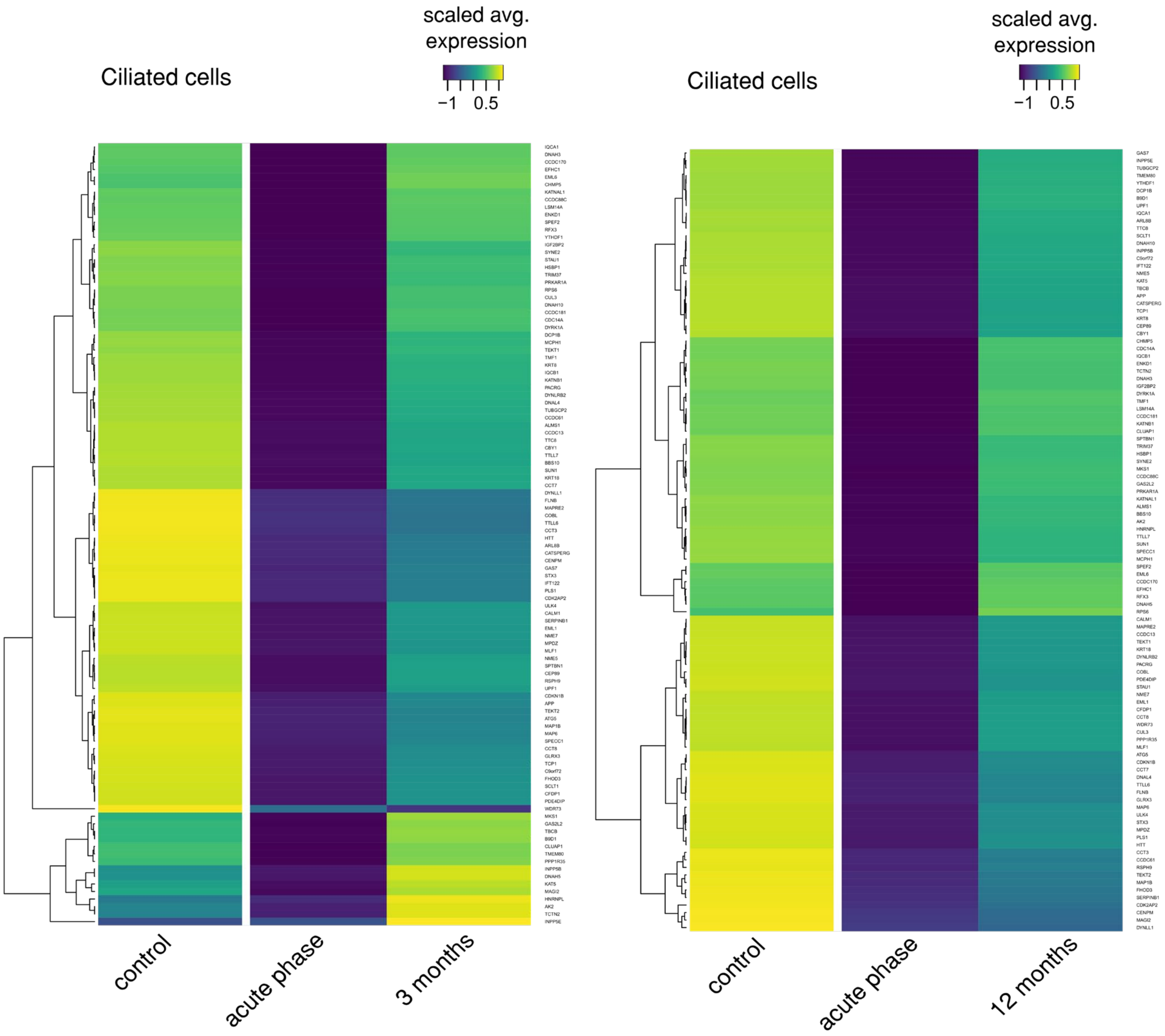

Appendix Figure S6: **a)** Heatmap left (short-term: 3 months follow-up): Controls (n=10), acute phase (n=6) and 3 months follow-up (n=6) only from those samples that have scRNA-seq data from acute phase and 3 months follow-up. **b)** Heatmap right (long-term: 12 months follow-up): Controls (n=10), acute phase (n=4) and 12 months follow-up (n=4) only from those samples that have scRNA-seq data from acute phase and 12 months follow-up. Those pathways score genes (n=104) that were downregulated in acute vs. control and 12 months vs. control in ciliated cells were used (= genes that were used for Cytoscape network in **Figure 4**).

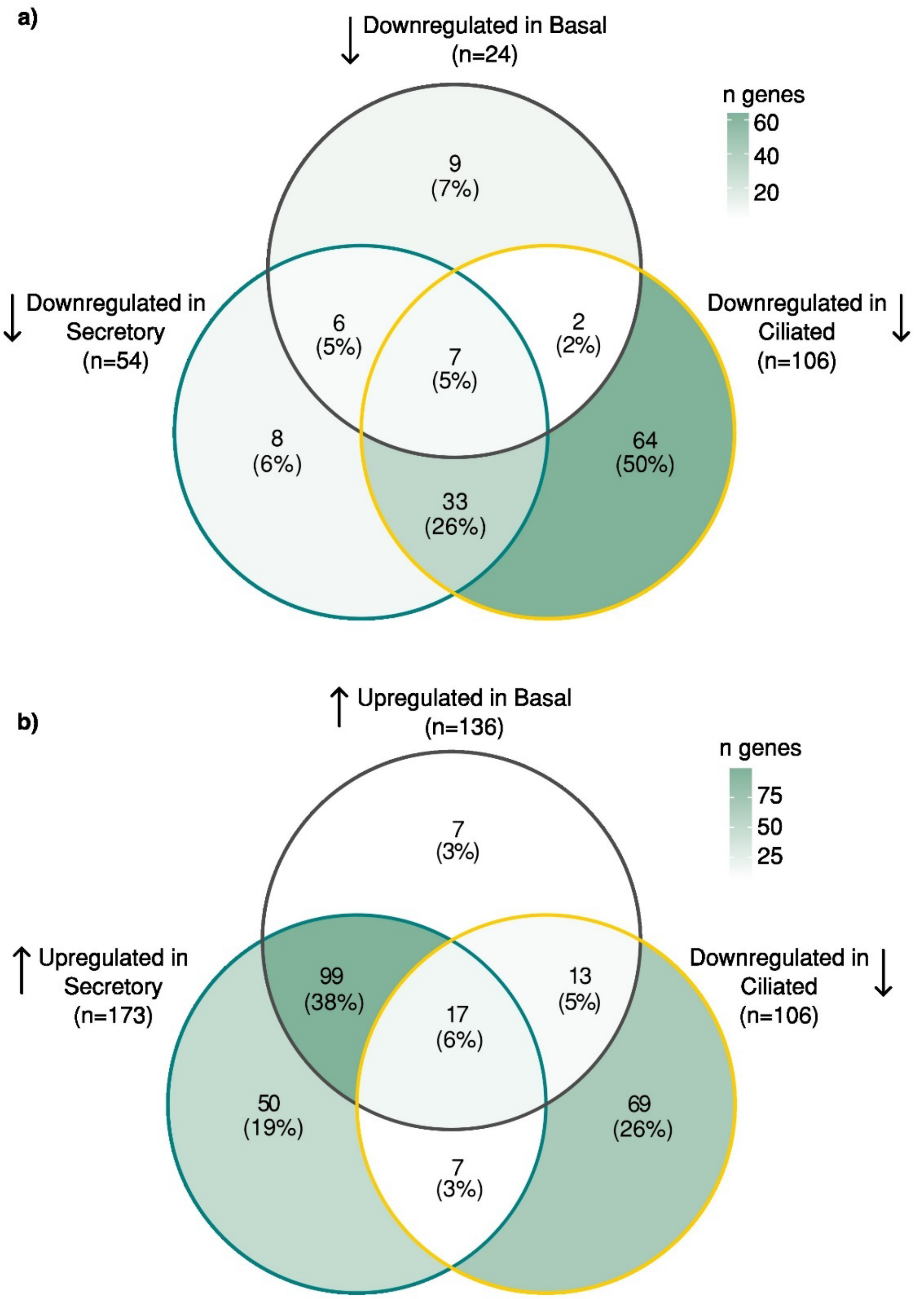

Appendix Figure S7: Venn diagrams showing overlaps between *pathways score*-derived differentially expressed genes comparing 12 months follow-up samples to controls. Differential gene expression was calculated using the MAST test, adjusted for age and proportion of genes per cell. **a)** Overlaps between genes that were downregulated in basal, secretory or ciliated cells. **b)** Overlaps between genes that were downregulated in ciliated cells and those that were upregulated in basal or secretory cells (**Dataset EV6**).

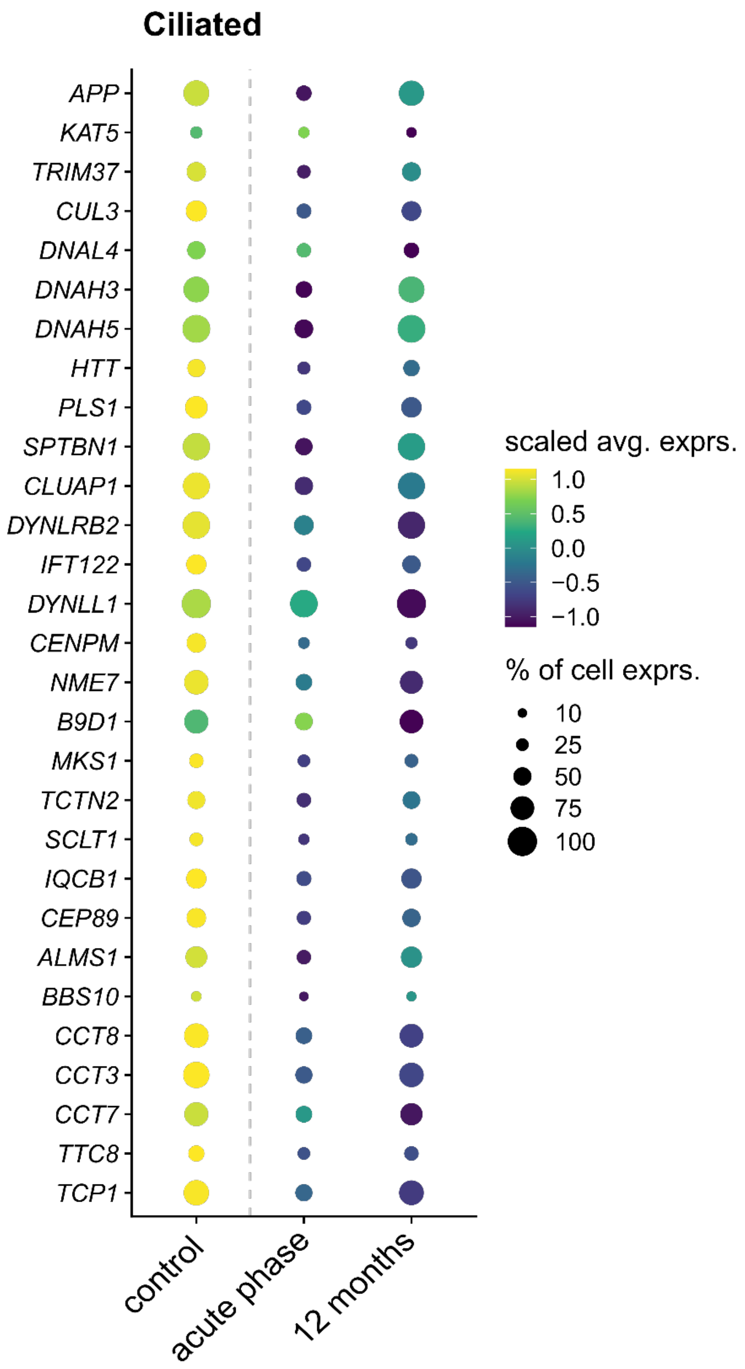

Appendix Figure S8: Dot plot depicting expression levels of all 30 genes from the Cytoscape network in **Figure 4c** in ciliated cells. All genes were significantly downregulated in the acute phase of infection as well as 12 months post infection compared to controls in ciliated cells (see **Dataset EV6**)

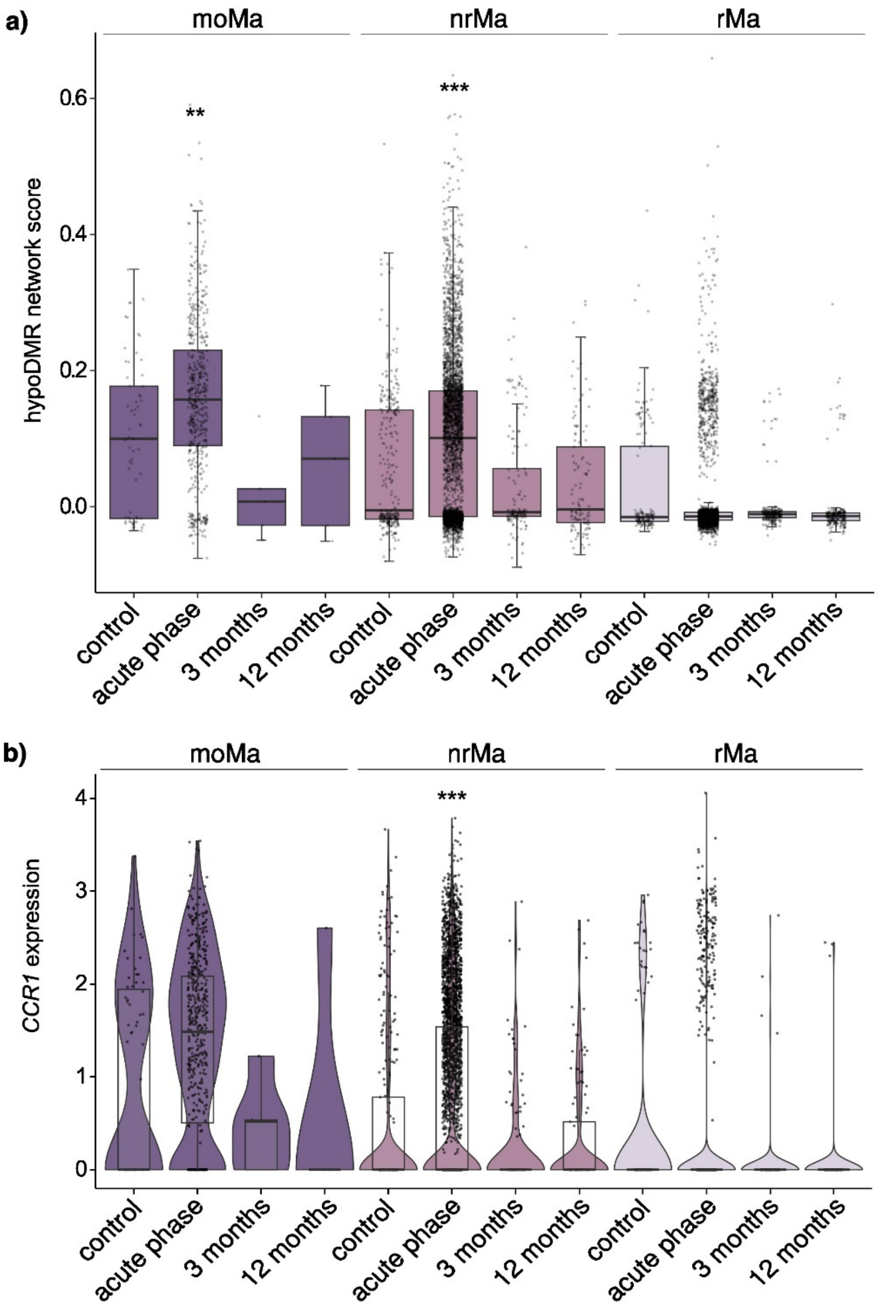

Appendix Figure S9: Expression time course of hypoDMR target genes. **a)** Boxplots depicting *hypoDMR network score* levels based on all hypoDMR target genes from the network in **Figure 2a**. **b)** Violin plots showing the expression levels of *CCR1* in macrophages. Significant increases in comparison to controls are indicated by asterisks, FDR-adj. *p* ** <0.01, *** <0.001. *HypoDMR network scores* were compared using Wilcoxon rank sum test (**Table EV10**), differential *CCR1* expression was determined using MAST, adjusted for age, proportion of genes per cell and sex, where applicable. Boxes indicate 25–75% quantiles, whiskers extend to 1.5 times of the interquartile range and the median is represented by a line.

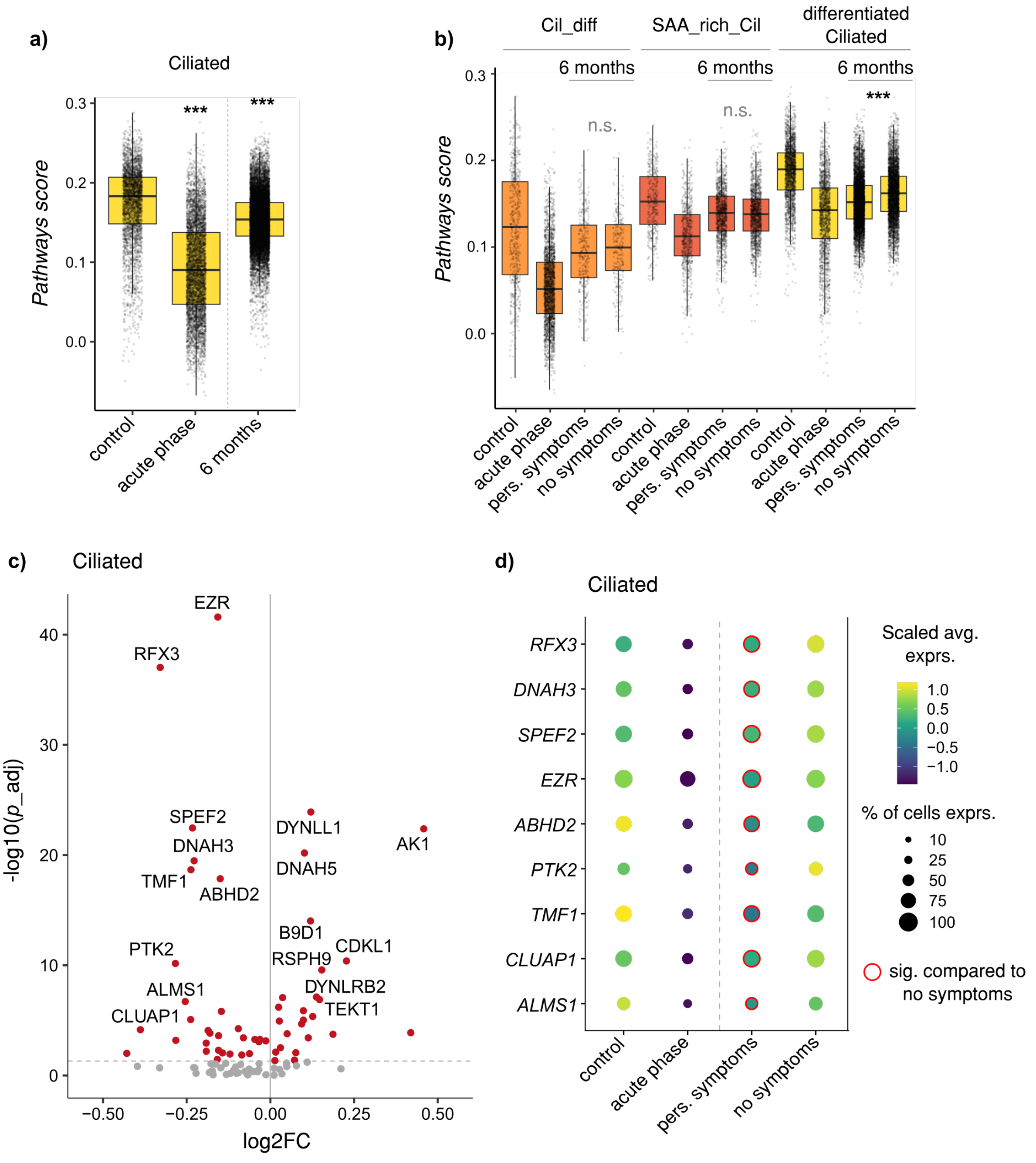

Appendix Figure S10: Expression of *pathways score* genes in independent samples collected 6 months post-infection (n=15) **a)** *Pathways score* levels in ciliated cells of controls, acute phase and 6 months follow-up samples. *Pathways score* values were compared against controls using multiple linear regression, adjusted for age, sex and the fraction of genes per cell. Acute phase vs. controls: coefficient = -0.047 (95% confidence interval [CI], -0.050 to -0.044), 6 months vs. controls: coefficient = -0.019 (95% CI -0.020 to -0.018). **b)** *Pathways score* levels in those cell types that form the ciliated cell group. *Pathways score* values between donors with (n=6) and without persisting respiratory symptoms (n=9) were compared using multiple linear regression, adjusted for age, sex and the fraction of genes per cell. Persisting symptoms vs. no symptoms in differentiated ciliated cells: coefficient = -0.006 (95% CI -0.007 to -0.004). Boxes indicate 25–75%

quantiles, whiskers extend to 1.5 times of the interquartile range and the median is represented by a line. **c)** Volcano plot showing all differentially expressed *pathways score* genes (adjusted for age, sex and fraction of genes per cell) between individuals with vs. without persisting respiratory symptoms (**Dataset EV7**). Red dots represent significantly differentially expressed genes (FDR-adjusted *p*-value <0.05). **d)** Dot plot depicting all genes involved in ciliary function that were significantly downregulated in donors with persisting respiratory symptoms vs. no symptoms. Cil_diff = differentiating ciliated cells, SAA_rich_Cil = SAA+ ciliated cells, *** FDR-adj. *p*-value <0.001
